# Selected rhizobacteria strains improved the tolerance of *Vicia faba* plants to microcystins contaminated irrigation water and reduced human health risk

**DOI:** 10.1007/s11356-025-37185-7

**Published:** 2025-11-21

**Authors:** Nadia Elidrissi El Yallouli, El Mahdi Redouane, Richard Mugani, Lahcen Ouchari, Mariana Girão, Maria Fátima Carvalho, Alexandre Campos, Vitor Vasconcelos, Brahim Oudra, Majida Lahrouni, John Poté

**Affiliations:** 1 Biotechnologies and Sustainability of Natural Resources Laboratory (Aquabiotech), Cadi Ayyad University of Marrakech, 40000 Marrakech, Morocco; 2 Faculty of Sciences, Department F.-A. Forel for Environmental and Aquatic Sciences and Institute for Environmental Sciences, University of Geneva, 1211 Geneva-4, Geneva, Switzerland; 3 Université de Reims Champagne-Ardenne, Université Le Havre Normandie, INERIS, Normandie Univ, UMR-I 02 SEBIO, BP 1039 51687 Reims, CEDEX Reims, France; 4 National Institute of Public Health, Ministry of Health, Avenue Ruvubu, BP 6807, Bujumbura, Burundi; 5 Molecular Biology and Functional Genomics Platform, National Center for Scientific and Technical Research (CNRST), 10000 Rabat, Morocco; 6 Microbiology and Molecular Biology Team, Plant and Microbial Biotechnology, Biodiversity and Environment Center, Faculty of Sciences, Mohammed V University in Rabat, 10000 Rabat, Morocco; 7 CIIMAR - Interdisciplinary Centre of Marine and Environmental Research, University of Porto, 4450-208 Porto, Portugal; 8 ICBAS - Institute of Biomedical Sciences Abel Salazar, University of Porto, 4050-313 Porto, Portugal; 9 Department of Biology, Faculty of Sciences, University of Porto, 4169-007 Porto, Portugal

**Keywords:** Microcystins, Bioremediation, Plant growth-promoting rhizobacteria, *Vicia faba*, Health risk

## Abstract

In Morocco as well as in many countries located in semi-arid areas, irrigation with water from lake-reservoirs is a common agricultural practice and an important tool for farmers to improve crop yields. However, this kind of water can contain toxic bloom-forming cyanobacteria, and its use as a source for irrigation water can enable a transfer of cyanobacterial toxins (microcystins) into crop plant. When microcystins are accumulated in crop plants, they pose serious human health risk. The aim of this study is to explore the potential of selected rhizobacteria strains in inoculation with *Vicia faba* plants to mitigate microcystins-induced phytotoxicity and health risks. Irrigation with water containing 200 µg L^−1^ microcystins reduced plant growth, photosynthetic efficiency, and nitrogen assimilation. Inoculation with selected rhizobacteria strains alleviated these effects, enhancing root biomass, stomatal conductance, chlorophyll content, leaf quantum yield, and nitrogen content and slightly increasing GS activity. Among tested strains, *Achromobacter marplatensis* showed the strongest protection, reducing microcystin accumulation by approximately 36% compared to uninoculated plants. Estimated daily intake values derived from residual microcystins remained below World Health Organization safety thresholds, indicating a lowered potential risk. These findings demonstrate that soil-based inoculation with targeted rhizosphere bacteria can protect plants from microcystin-induced damage, providing a strain-specific, sustainable bioremediation strategy to maintain crop productivity and food safety in regions exposed to microcystin-contaminated irrigation water.

**Supplementary Information:**

The online version contains supplementary material available at 10.1007/s11356-025-37185-7.

## Introduction

The rise of temperature induced by climate change and the eutrophication of aquatic ecosystems create conditions favorable for the proliferation of toxic cyanobacteria, which produce toxins (cyanotoxins). Microcystins (MCs) are the most prevalent cyanotoxins due to their hepatotoxicity and widespread occurrence (Krishnan & Mou 2021; Mokoena 2024; Zhao et al. 2024). MC-LR variant is the most toxic among MC analogs and serves as a reference for toxicity and concentration, followed by MC-RR and MC-YR (Ma et al. 2021). These compounds are synthesized by living cyanobacteria and mainly released by membrane diffusion or after cell death (Pantelić et al. 2013; Tanvir et al. 2021). Consequently, these toxins may be found in high concentrations in aquatic ecosystems, leading to adverse effects on crop product quality when contaminated water is used for irrigation (Haida et al. 2022; Redouane et al. 2023; Faulkner et al. 2025). It has been estimated that the half-life of the MC-LR in agricultural soil is approximately between 6 to 56 days due to its cyclic structure, while the photosensitized degradation of the toxin in aquatic ecosystems takes around 90–120 days per meter depth of the water column, potentially leading to food chain contamination and posing risks to human health (Chen et al. 2006; Corbel et al. 2014, 2016; Morón-López & Molina 2020). Therefore, the persistence of these toxins in both irrigation water and soil can significantly impact the growth and development of cultivated plants, resulting in significant economic losses (Corbel et al. 2016).

The eutrophic semi-arid Lalla Takerkoust lake-reservoir, located at 35 km southwest of Marrakesh city, Morocco, is a vital freshwater resource supporting agricultural irrigation in the Marrakech region (Gourfi & Daoudi 2019). This reservoir exemplifies the persistence and impacts of toxic cyanobacterial blooms, particularly those dominated by *Microcystis aeruginosa* during the summer-autumn seasons, with environmental concentrations of dissolved MCs in irrigation water ranging from 95 to 160 μg L⁻^1^ (Mugani et al. 2024). Among the crops irrigated with this MC-contaminated water is faba bean (*Vicia faba L*.), a leguminous species of major agronomic significance in Morocco’s cropping systems. This is due to its position in crop rotation, where it follows cereal, and to their economic and nutritional importance. *V. faba* has the ability to bolster soil nitrogen levels and reduce reliance on nitrogen fertilizers. This is attributed to nitrogen fixation through symbiotic processes with rhizobia nodules (Luce et al. 2013). However, exposure to MC-contaminated irrigation water threatens these benefits, as MCs disrupt plant physiological processes, leading to reduced growth, impaired photosynthesis, and lower nitrogen assimilation, ultimately compromising yield and soil fertility (Faulkner et al. 2025).

Beyond agricultural losses, MC bioaccumulation in *V. faba* raises food safety concerns due to potential transfer into human and livestock food chains (Melaram et al. 2022; Faulkner et al. 2025). Conventional MC removal methods including adsorption, chlorination, photocatalysis, and oxidation are costly, incomplete, and may generate secondary pollution (Ding et al. 2022; Zhan & Hong 2022). Microbial bioremediation using rhizobia or rhizobacteria offers a cost-effective, eco-friendly alternative, yet its application in soil-grown legumes remains scarcely addressed (Bianco 2024; Chandrasekaran & Paramasivan 2024).

Several studies have examined microbial mitigation of MC stress, but their systems differed in contamination levels, plant hosts, and microbial strategies, limiting transferability to soil agriculture. Lahrouni et al. (2016) showed that inoculation with *Rhizobium* strains protected nitrogen metabolism in *V. faba* at 100 μg L^−1^ MCs in sand-vermiculite, but this excluded soil microbial interactions. Haida et al. (2024) demonstrated that *Shinella* and *Ensifer* degraded MCs and reduced bioaccumulation in hydroponically grown strawberry (*Fragaria vulgaris*), but only at 10–20 μg L^−1^, far below concentrations reported in Moroccan reservoirs. In contrast, Redouane et al. (2021a) found that native rhizospheric microbiota in agricultural soils mitigated phytotoxicity and reduced MC accumulation in *V. faba* under exposures up to 2.5 mg L^−1^, while Redouane et al. (2021b) confirmed protection in non-sterile soils at 100 μg L^−1^ in both *V. faba* and wheat. However, these protective effects relied on indigenous consortia without targeted inoculation, making outcomes variable and difficult to standardize. Collectively, these findings indicate that while rhizobia or rhizospheric microbes can alleviate MC stress, targeted strategies combining selected toxin-tolerant rhizobia and rhizobacteria with growth-promoting traits under soil conditions and environmentally relevant high MC exposure remain largely unexplored.

This study is aimed at prospecting the potential effectiveness of MC-resistant rhizobia and rhizobacteria exhibiting plant growth-promoting traits to improve *V. faba* plants responses under MC-stress by integrating of these microorganisms in plant-soil system as biocontrol agent against MCs. These responses include growth parameters (plant height, leaf number, and plant dry weight), photosynthetic parameters (stomatal conductance, leaf quantum yield, pigment content), nitrogen assimilation (nitrogen content and glutamine synthetase activity), and MC-bioaccumulation in plant tissues. Special interest will be given to rhizobia strains because of their ability to make association with legume plants roots to provide nitrogen. The hypothesis that underlies this research work is the natural ability of soil microorganisms to ensure proper performance in the absence of nitrogen fertilization and under MCs stress. Our aim is to prospect beneficial legume-bacteria interactions to cope with MC-stress in the rhizosphere before the toxins reach the food chain. We are projecting to improve crop production, agriculture, and food security.

## Materials and methods

### Cyanobacterial bloom

In October 2018, a cyanobacterial bloom sample was collected from the Lalla Takerkoust lake-reservoir (31° 36′ N, 8° 2′ W, 664 m) near Marrakesh, Morocco, using a 27-µm mesh phytoplankton net. Microscopic analysis identified *M. aeruginosa* as the dominant species, representing approximately 75.3% of the total cell population. UPLC-MS/MS quantification indicated a total MC concentration of 14.67 μg mL⁻^1^ in the fresh bloom. The limit of quantification (LOQ) was estimated for a signal-to-noise (S/N) ratio equal to 10 from the chromatograms of the samples spiked at the lowest validated level, while the limit of detection (LOD) was calculated for *S*/*N* = 3 from the same chromatograms. Chromatographic analysis distinguished three MC variants: MC-YR (53.47%), MC-LR (44.18%), and MC-RR (2.35%) (Redouane et al. 2024).

### Cyanobacterial aqueous crude extract preparation and microcystins pre-purification

Following the method described by Saqrane et al. (2009) with slight modifications, bloom material predominantly composed of *M. aeruginosa* underwent ultrasonication in an ice bath to avoid MC degradation caused by heat from the sonication process. The lysate was subsequently centrifuged at 4000 × g for 15 min. To enhance purification, the supernatant was re-centrifuged under the same conditions, repeating this step twice to ensure effective removal of cellular debris. The resulting supernatants from all steps were then combined to obtain a clarified extract. To concentrate and purify the MCs, solid-phase extraction was employed. Octadecyl-silica cartridges (LiChrolut® RP-18, particle size 40–63 µm, capacity 1000 mg/6 mL, purchased from Sigma-Aldrich, USA) were conditioned by sequentially passing 5 mL of pure methanol (≥ 99%) and 5 mL of ultrapure water. The extract was then loaded onto the cartridges, and non-specifically bound compounds were removed by washing with 10 mL of 20% aqueous methanol (v/v). The retained MCs were eluted using 70% methanol (v/v). The eluate was subsequently vacuum-dried at 40 °C, reconstituted in 1 mL of ultrapure water, and stored at − 20 °C for future use. This extract served as a stock solution to prepare MC-enriched irrigation water at a final concentration of 200 µg MC equivalent L⁻^1^. The contaminated water was later used for irrigating *V. faba* plants in the greenhouse experiment.

### Rhizobacterial strain isolation from agricultural soil

Fifteen rhizobacterial strains (including rhizobia) were isolated from agricultural fields in the area surrounding Lake Lalla Takerkoust, Marrakech, Morocco. Rhizobacterial strains T2, T3, T4, T9, T10, F7, F9, and B9 were isolated from the rhizospheres of *Trifolium alexandrinum*, *Triticum aestivum*, and *V. faba*. Additionally, rhizobial strains RN1, RN2, RN3, RN4, RN5, RN6, and RN7 were isolated specifically from the root nodules of *V. faba* plants within the same region.

### Isolation of rhizobacterial strains

Isolation of rhizobacterial strains (T2, T3, T4, T9, T10, F7, F9, and B9) was performed using an enrichment method in liquid mineral salt medium (MSM), which consisted of 4 g of K₂HPO₄, 0.5 g of KH₂PO₄, 0.5 g of (NH₄)₂SO₄, 1 g of NaCl, 23 mg of CaCl₂.H₂O, 1 g of MgSO₄0.7H₂O, 0.01 g of FeSO₄0.7H₂O, 0.005 g of MnCl₂0.4H₂O, 0.005 g of ZnCl₂, and 300 µL of CuCl₂0.2H₂O per liter of distilled water. The medium was supplemented with MCs as the sole carbon and nitrogen source. This approach ensured the selective enrichment of bacteria capable of MC utilization. Strains unable to grow in the MSM medium were systematically excluded (Shen et al. 2019).

### Isolation and purification of rhizobial strains

Rhizobial strains (RN1, RN2, RN3, RN4, RN5, RN6, and RN7) were isolated from *V. faba* root nodules. Nodules were surface-sterilized (15 min in sodium hypochlorite 2°), rinsed several times with sterile physiological water (0.9% NaCl), and crushed in 1 mL sterile water. The resulting suspension was serially diluted and plated on Yeast Extract Mannitol (YEM) medium. After 48 h at 28 °C, colonies with a gluey appearance and no Congo red absorption were isolated and purified on YEM medium (Vincent 1970).

### Screening rhizobial strains for microcystin utilization on solid mineral salt medium agar

Rhizobial strains were screened for their ability to use MC as the sole source of carbon and nitrogen on MSM agar. The pH was adjusted to 7 using a laboratory pH meter, and the medium autoclaved at 121 °C for 20 min. The medium was then supplemented with 2 mg/L of pre-purified MCs. Strains were streaked onto Petri dishes containing the supplemented medium and incubated at 28 °C ± 2 °C for up to 3 days with three replicates. Negative control plates without MCs were included to ensure that observed growth was specifically due to the utilization of MCs. Growth on this medium indicated the strains’ potential for MC biodegradation (Zhang et al. 2017). The results indicated that the rhizobacterial strains T2, T3, T4, T9, T10, F7, F9, B9, RN1, RN2, RN5, and RN7 could grow in mineral medium supplemented with MCs as sole carbon and nitrogen sources (MSM medium) (SI: Table S1). This means that these strains have the ability to utilize MCs for their growth. However, in the same medium, the rhizobacterial strains RN3, RN4, and RN6 exhibited no growth at all (SI: Table S1), which means that they could not use MCs as carbon and nitrogen sources for their growth. It is also important to note that these three strains (RN3, RN4, and RN6) were able to grow in nutrient agar medium (composition: peptone 5 mg, beef extract 3 mg, sodium chloride 5 mg, agar 15 mg). This result confirms that these three strains are not able to deal with MCs; thus, they will not be used in plant-soil system as bioremediation agents.

### Plant growth-promoting activity screening

Selected strains, which demonstrated the ability to utilize MC as the sole source of carbon and nitrogen, were screened for PGPR activities. This included assays for phosphate solubilization (PS), nitrogen fixation, hydrogen cyanide production (HCN), indole 3-acetic acid (IAA) synthesis, and protease and chitinase activities. All experiments were conducted in triplicate.

### Indole3-acetic acid production assay

IAA production was quantified for selected strains. Bacterial suspensions (100 µL, OD₆₀₀ = 0.8, *n* = 3) were grown in 10 mL TSB with 0.1% L-tryptophan at 28 ± 2 °C (200 rpm) for 4 days. Cultures were centrifuged (5000 rpm, 10 min, 4 °C), and IAA in the supernatant was estimated colorimetrically. Salkowski reagent (1 mL; 0.5 M FeCl₃, 30% perchloric acid) was added to 1.5 mL of supernatant and incubated in the dark (30 min, 26 ± 2 °C). A pink color indicated IAA presence, measured at 530 nm (Cary 50 Scan, Australia). IAA concentration (µg mL⁻^1^) was calculated from a standard curve using commercial IAA (Sigma-Aldrich) (Mahdi et al. 2021).

### Phosphorus solubilization

The qualitative estimation of phosphorus solubilization for the selected strains was conducted on a phosphate growth medium composed of 10 g glucose, 5 g Ca_3_(PO_4_)_2_, 0.5 g (NH_4_)_2_SO_4_, 0.2 g NaCl, 0.1 g MgSO_4_.7H_2_O, 0.2 g KCl, 0.002 g MnSO_4_.H_2_O, 0.002 g FeSO_4_.7H_2_O, and 15 g agar in distilled water. After 10 days of incubation, the solubilization index (SI) was determined using the formula: SI = diameter of the solubilization zone divided by the diameter of the colony (Alikhani et al. 2006).

### Qualitative analysis of nitrogen fixation

The nitrogen-fixing potential of bacterial strains was determined by their growth on N-free Jensen’s medium after incubation at 30 ± 2 °C for 7 days. Growth on this medium served as an indicator of their capacity for atmospheric nitrogen fixation (Sherpa et al. 2021).

### Hydrogen cyanide production assay

HCN production was assessed using the method of Cherif-Silini et al. (2019). Bacteria were grown on HCN medium (TSB 30 g/L, glycine 4.4 g/L, agar 15 g/L). A Whatman® filter soaked in 0.5% picric acid and 2% Na_2_CO_3_ was placed on the plate lid and then sealed with parafilm. After 6 days at 28 ± 2 °C, an orange-brown color indicated HCN production. Experiments were performed in triplicate.

### Production of protease

Proteolytic enzyme production was evaluated by inoculating pure colonies of each bacterium on skim milk agar plates (Ali et al. 2020). The plates were then incubated at 28 ± 2 °C for 5 days. The presence of a clear zone around the bacterial colony indicated positive enzyme activity.

### Evaluation of microbial strains for plant growth promotion

The rhizobacterial strains (T2, T3, T4, T9, T10, F7, F9, B9, and RN7) showing a strong growth in MSM medium (SI: Table S1) were subject to the evaluation of their PGPR traits (Table 1). T4, T10, and RN7 strains exhibited at least three positive PGPR traits (Table 1), making them strong candidates for further research. However, the other rhizobacterial strains (T2, T3, T9, F7, F9, and B9) showed a maximum of two positive PGPR traits (Table 1); thus, they will not be used in plant-soil-system as bioremediation agents.

**Table 1:** Plant growth promoting traits of rhizobacterial strains

Isolates	Plant growth promoting traits			Biocontrol traits	
	PS (SI)	N	IAA production (μg/mL)	HCN	Protease (SI)
	10 days	7 days	4 days	6 days	5 days
T4	1.22 ± 0.19	+	184.5 ± 0.10	+	-
T10	1.91 ± 0.53	+	13.02 ± 0.01	+	-
T2	−	−	−	−	1.91 ± 0.14
T3	−	−	−	−	1.76 ± 0.09
T9	−	+	−	−	−
B9	−	+	52.89 ± 0.00	−	−
F7	1.35 ± 0.06	+	−	−	−
F9	−	+	−	−	1.50 ± 0.17
RN7	−	+	19.82 ± 0.00	−	2.57 ± 0.53

*IAA*, indole acetic acid; *PS*, phosphate solubilization; *SI*, solubilization index; *N*, nitrogen fixation; *HCN*, hydrogen cyanide. The (+/−) indicates positive and negative results respectively. Values are mean of three independent observations

The three rhizobacterial strains (T4, T10, and RN7) demonstrating strong growth in the presence of MCs and exhibiting at least three positive PGPR traits (SI: Table S1 and Table 1) were subject to molecular identification, and they will be used alone and in consortium in soil plant-system as a bioremediation agent.

### Molecular identification of the selected bacterial isolates and phylogenetic analysis

**Fig. 1:**
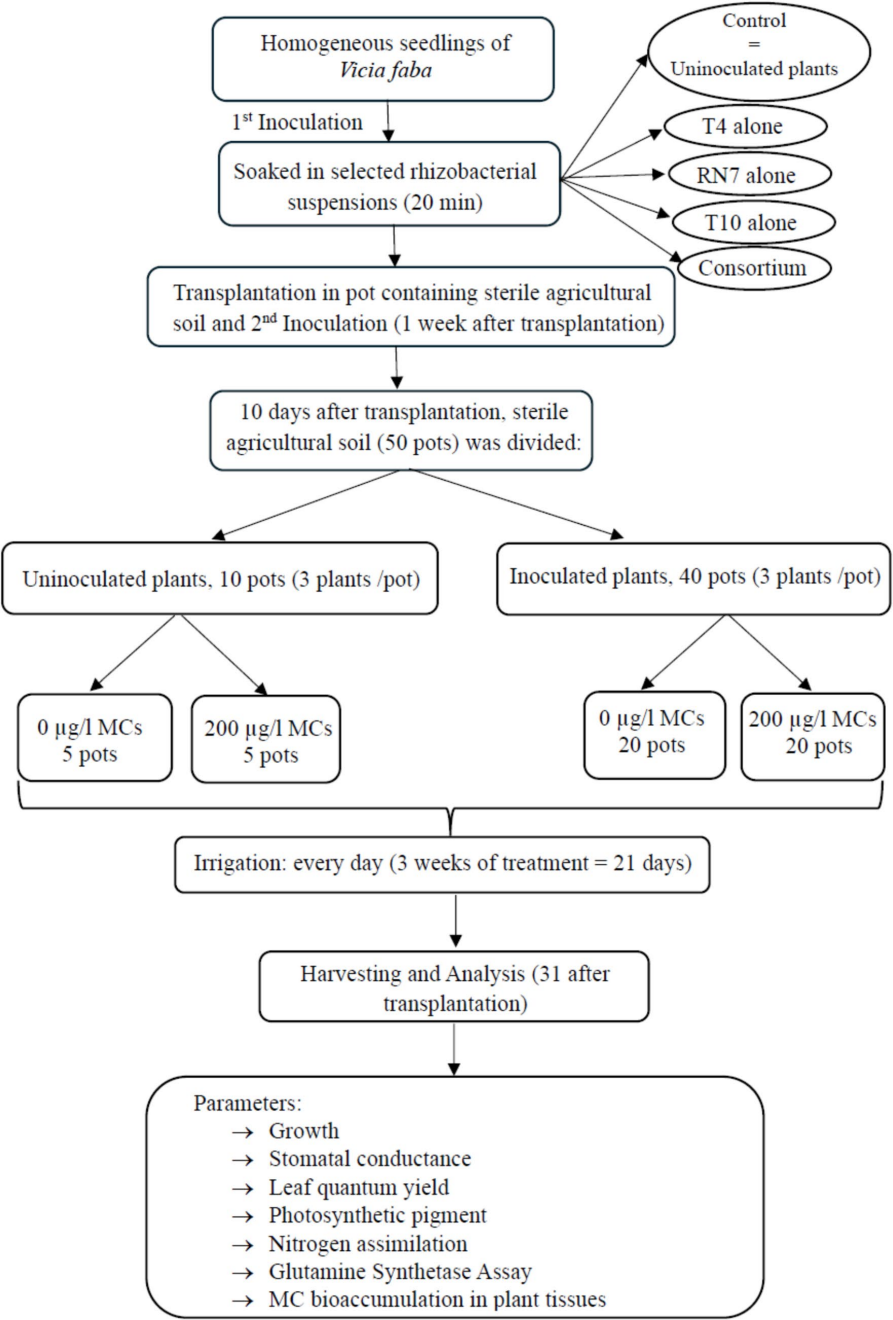
Greenhouse experimental design for evaluating plant growth promoting rhizobacteria effects on *Vicia faba* plants under microcystin stress

**Fig. 2:**
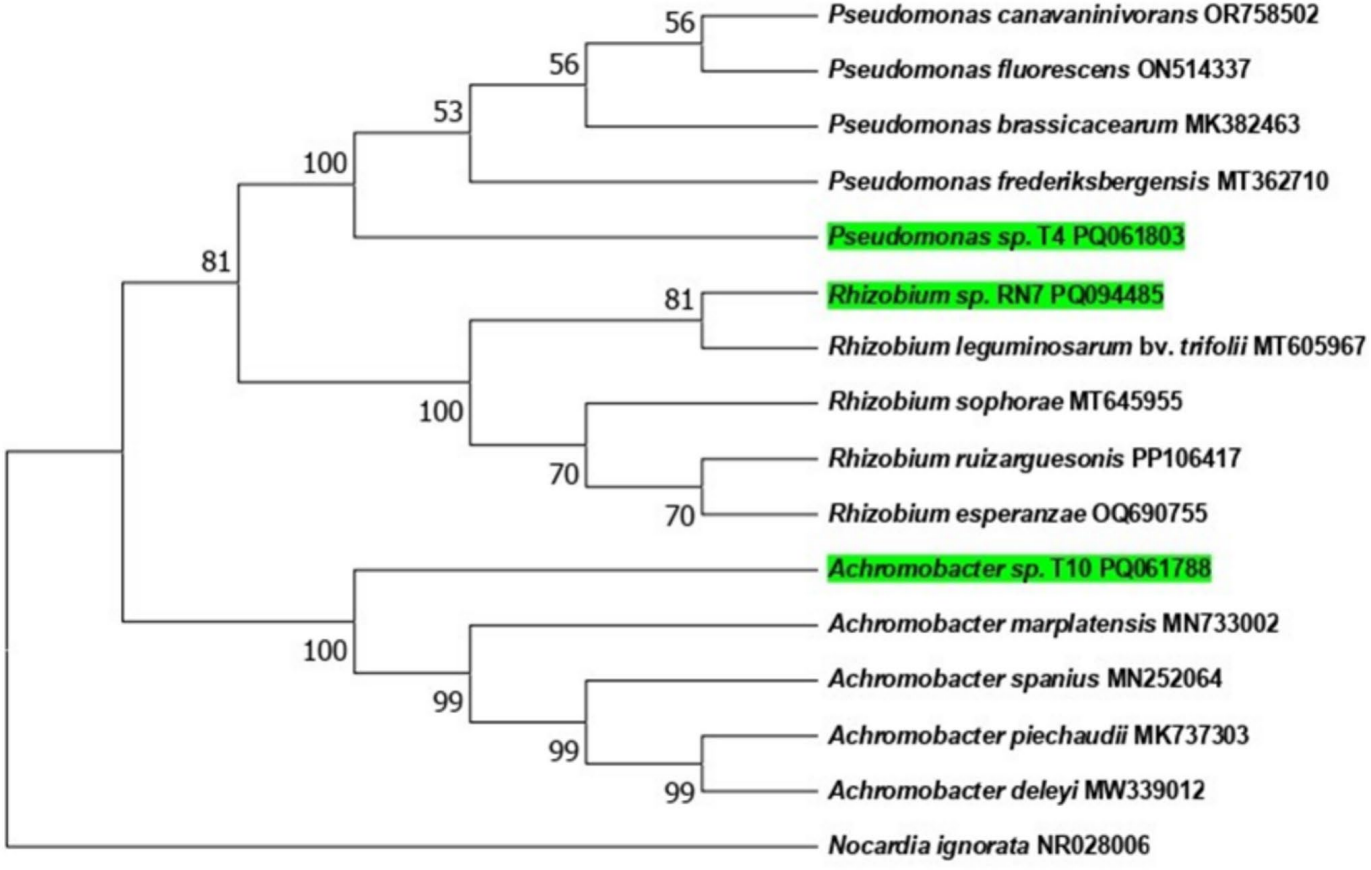
Phylogenetic tree of T4, T10, and RN7 strains (highlighted in green) and their nearest clustering neighbors. Evolutionary history was inferred using the neighbor‐joining method. Bootstrap values were converted to percentages, and the branches corresponding to the partitions replicated in less than 50% of the bootstrap replicates were removed. The percentage of replicate trees in which the associated taxa clustered together in the bootstrap test (1000 replicates) are shown next to the branches (Felsenstein, 1985). *Nocardia ignorata* (NR028006) serves as the outgroup

Taxonomic identification of all isolates was carried out via 16S rRNA gene sequencing. For DNA extraction, bacterial biomass was obtained after 24 h of incubation on Luria–Bertani agar medium. The collected biomass was transferred into a tris-buffer solution using sterile disposable loops. Genomic DNA was extracted using the E.Z.N.A Bacterial DNA Kit (Omega Bio-Tek, based in Georgia, USA). PCR amplification of the 16S rRNA gene was performed using the universal primers 27F/1492R (Weisburg et al. 1991), following the methodology outlined by Girão et al. (2019). PCR products were purified using the ExoSAP-IT (TM) Express kit (Applied Biosystems, based in Warrington, UK), which includes a one-step enzymatic treatment to remove any unincorporated primers and dNTPs, following the manufacturer’s protocol. The purified DNA (2.5 mL) was then sequenced using the Genetic Analyzer 3130xl sequencer (Applied Biosystems, Warrington, UK), according to the manufacturer’s guidelines. The raw DNA sequences were analyzed, end-trimmed, and aligned using SeqTrace, version 0.9.0. (Stucky 2012). The partial 16S rRNA gene sequences of the isolates were submitted to GenBank (NCBI, Bethesda, MD, USA) and assigned the following accession numbers: PQ061788 for T10, PQ061803 for T4, and PQ094485 for RN7. Evolutionary analyses were conducted in MEGA11 (Tamura et al. 2021). The evolutionary history was inferred using the neighbor-joining method (Saitou et Nei, 1987). The phylogenetic tree was constructed to scale, with branch lengths representing the evolutionary distances that were used to infer the tree’s structure. These distances were calculated using the Maximum Composite Likelihood method (Tamura et al. 2004). This analysis involved 16 nucleotide sequences. All ambiguous positions were removed for each sequence pair (pairwise deletion option). There were a total of 1516 positions in the final dataset. *Nocardia ignorata* (NR028006) was used as an outgroup to construct the phylogenetic tree (Fig. 1). The molecular identification, based on 16S rRNA gene sequencing, classified them as *Achromobacter* sp., *Pseudomonas* sp., and *Rhizobium* sp., related to *Achromobacter marplatensis*, *Pseudomonas frederiksbergensis*, and *Rhizobium leguminosarum* bv. *trifolii*, respectively (Fig. 2).

### Root colonization bioassay

This assay assessed the ability of selected bacterial strains to colonize *V. faba* roots. Seeds were surface-disinfected (2% NaOHCl, 17 min) and rinsed with sterile water. Seedlings were soaked for 24 h in 5 mL bacterial suspensions of (1) strain T4, (2) strain T10, or (3) control and then transferred to sterile 0.6% water-agar tubes. Root-associated bacterial growth was visually inspected daily (Silva et al. 2003). The results demonstrated that the most effective strains (T4, T10, and RN7) were capable of colonizing *V. faba* roots. Upon eye inspection of the tubes and subsequent examination using a stereoscopic microscope, the roots exhibited turbid zones all around.

### Bacteria co-culture competition assay

Antagonism between strains T4, T10, and RN7 was evaluated using the line-streak assay. Each strain was streaked onto YEM medium (three replicates) and incubated for 48 h. Cross-streaks of test bacteria were then added and incubated for another 48 h at 28 ± 2 °C. Growth inhibition along the new streak indicated antagonistic activity (Khan et al. 2022). The results indicated that all selected strains were compatible for use as a consortium, as no growth inhibition zone was observed at the intersection areas (SI: Table S2). Therefore, these three strains were utilized as a consortium in *V. faba* culture to maximize PGPR traits.

### Greenhouse experiment

Three promising PGPR strains, along with one rhizobium strain, were selected based on their tolerance to MC stress and high PGPR activity. A greenhouse experiment was conducted at Cadi Ayyad University to evaluate the effects of these strains on *V. faba* plants under MC stress. The experiment followed a randomized complete design with five replicates per treatment. Environmental conditions included a temperature of 25.5 °C, 68.5% humidity, and a photon flux density of 410 μm^−2^.s^−1^.

*V. faba* (var. Alfia) seeds were surface-disinfected, germinated for 4 days, and inoculated with bacterial suspensions of strain T4, T10, and RN7 or a consortium of T4, T10, and RN7. Control plants were treated with sterile physiological water. After inoculation, seedlings were transplanted into sterile agricultural soil and re-inoculated 1 week later (Fig. 1).

During greenhouse experiment, in the first 10 days, all plants were irrigated with distillated water (adaptation phase). Then, plants were divided into two groups: control (irrigated with MC-free water) and treatment (irrigated with MC-contaminated water containing 200 µg L^−1^ MC-LR). Irrigation continued for 21 days; then, plants were harvested to assess growth, stomatal conductance, leaf quantum yield, pigment content, nitrogen assimilation, and MC bioaccumulation in tissues (Fig. 1).

### Growth parameters

After 3 weeks of exposure to cyanobacterial crude extract (200 µg L⁻^1^ MCs), plant height (cm) and leaf number were measured before harvest. The plants were divided into two groups: the first group (20 plants) shoots and roots were separated and then dried at 70 °C for 72 h to determine the dry weights. The second group (30 plants) shoots and roots were separated, frozen in liquid nitrogen, stored at − 20 °C, and freeze-dried for further analysis.

### Photosynthetic parameters

#### Stomatal conductance

Stomatal conductance (gs) was measured using a portable Porometer (Leaf Porometer, Decagon Devices, USA) between 10 am and 12 pm, during peak stomatal opening. Measurements were taken on the lower surface of healthy, young leaves from three plants per treatment. The parameter was expressed in mmol H₂O m⁻^2^ s⁻^1^ (Zeppel et al. 2012).

#### Leaf quantum yield

Leaf quantum yield was measured with a portable fluorometer (OPTI-sciences OSI 30p). Clips were applied to the upper surface of young leaves (same rank) with three repetitions per treatment. After 20 min of dark adaptation, minimal (*F₀*), maximal (*Fₘ*), and variable (*Fᵥ*) fluorescence emissions were recorded. The PSII efficiency was determined by the *Fᵥ*/*Fₘ* ratio (Li et al. 2006).

#### Photosynthetic pigment content

After 3 weeks of exposure to cyanobacterial extract, chlorophyll a (Chl a) and b (Chl b) contents were measured to assess the impact of MCs on photosynthetic pigments (Geider & Osborne 1992). Leaves (50 mg) were homogenized in 2 mL of 90% acetone, centrifuged, and extracted twice with 0.5 mL acetone. Chl a and Chl b concentrations were determined by absorbance at 664 and 647 nm using the equations:

- Chl a = (13.7*DO664nm)—(5.76*DO647nm).
- Chl b = (25.8*DO664nm)—(7.6*DO647nm).

### Nitrogen uptake analysis

#### Nitrogen content

Nitrogen content in roots and shoots was determined using the Kjeldahl method (Rodier et al., 1984). Dried plant material was digested with sulfuric acid and a catalyst mixture, distilled with NaOH, and the ammonia released was quantified by titration with sulfuric acid.

#### Glutamine synthetase assay

Leaf samples (200 mg) were ground in 1 mL extraction buffer 25 mM 3-(N-morpholino) propanesulfonic acid (MOPS), pH 7, 10 mM MgCl₂, 10% glycerol, 1 mM dithiothreitol (DTT), 0.1% β-mercaptoethanol, and 0.05% phenylmethylsulfonyl fluoride (PMSF) (Sigma-Aldrich). After centrifugation (5000 rpm, 15 min, 4 °C), the supernatant was used for GS activity and protein measurements.

GS activity was measured as described by Teixeira et al. (2005) with a 1 mL reaction mixture containing 120 μmol MOPS buffer (pH 7), 90 μmol L-glutamine, 2.4 μmol MnCl₂, 50 nmol ADP, 120 μmol hydroxylamine chloride (neutralized with 60 μmol NaOH), 50 μmol AsO₄^3^⁻, and crude extract. The mixture was incubated at 30 °C for 20 min, and absorbance at 500 nm was measured after forming a brown complex with FeCl_3_. The specific GS activity was expressed in mU mg protein⁻^1^.

GS activity:

- (A500nm*1000)/(1.08 × incubation time (min) × assay volume of the enzymatic extract in mL).
- 1.08: constant.
- Proteins = A595nm/(35 × assay volume of the enzymatic extract in mL).
- 35: constant.
- Therefore, the specific activity of GS in mU/mg: (GS activity in mU/mL)/(proteins in mg/mL).

#### Microcystins bioaccumulation in plant tissues

Total MCs in plant tissues were quantified using enzyme-linked immunosorbent assay (ELISA) with a microcystins/nodularins (ADDA) kit (520,011, Eurofins Abraxis, Warminster, PA, USA). Plant extracts were prepared as described by Saqrane et al. (2009), with slight modifications. *V. faba* leaves (250 mg) were homogenized with 10 mL of 70% aqueous methanol. After sonication (5 min) and centrifugation (5000 × g, 4 °C, 15 min), the supernatants were pooled, concentrated by rotary evaporation at 45 °C, and resuspended in 1 mL of ultrapure water for ELISA analysis as per the manufacturer’s instructions.

#### Health risk assessment

The health risks associated with consuming *V. faba* irrigated with MC-contaminated water were assessed through the estimated daily intake (EDI) and health risk quotient (RQ). EDI was calculated using the formula (Cordeiro-Araújo et al. 2016): EDI = (*C*_shoot_ × *D*_food intake_)/*B*_average weight_.

*C*_shoot_ represents the concentration of MCs in the plant shoots (µg/kg dry weight), *D*_food intake_ is the daily intake of *V. faba* (1.29 g), and *B*_average weight_ is the average body weight (60 kg) (FAO 2022). The RQ, which indicates the health risk, was determined by dividing EDI by the acceptable TDI set by the World Health Organization (WHO) (TDI, 0.04 μg kg^−1^). Risk levels were classified as follows: RQ > 1 (high), 0.1 ≤ RQ ≤ 1 (moderate), and RQ < 0.1 (low) (Gu & Liang 2020).

#### Statistical analysis

Statistical analyses and data processing were carried out using Minitab 19. To assess the effects of MC on each parameter and the impact of bacterial treatments, a two-sample *t*-test and a one-way ANOVA with the Fisher test were employed, respectively. Statistical significance was set at *p* ≤ 0.05, indicating significant differences between treatments.

## Results

### Growth responses of *Vicia faba* to microcystin exposure and rhizobacterial inoculation

**Fig. 3:**
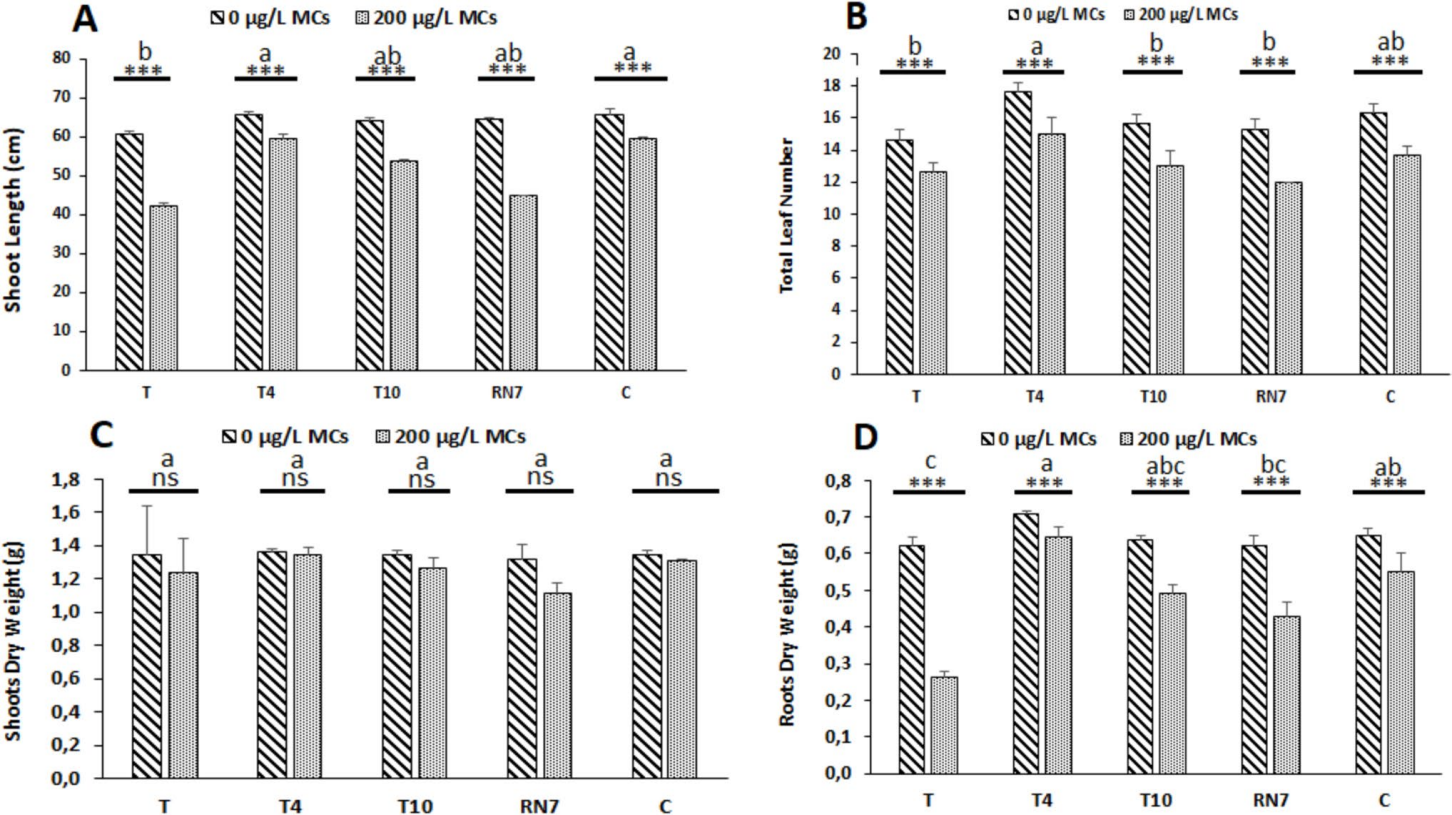
Effects of cyanobacterial crude extract containing 200 µg.L^−1^ of microcystins on *Vicia faba* growth after 3 weeks of exposure: **A** shoot length (SL) in cm, **B** total leaf number (TLN), **C** shoot dry weight (SDW) in g, and **D** root dry weight (RDW) in g under various treatments: T: control (uninoculated plants); T4, T10, and RN7: plants inoculated with rhizobacterial strains; C: plants inoculated with a consortium of rhizobacterial strains (T4 × T10 × RN7). Mean values (± standard deviations) derived from three replicates. The stars correspond to statistical significance at different levels as follows: ns, not significant; * = *p* < 0.05; ** = *p* < 0.01; *** = *p* < 0.001. Means not sharing the same letter are considered significantly different

Three bacterial strains (T4: *Achromobacter marplatensis*, T10: *Pseudomonas frederiksbergensis*, RN7: *Rhizobium leguminosarum* bv. *trifolii*) were selected for MC metabolism and multiple growth-promoting traits (Tables 1 and S1; Fig. 2). Compatible in co-culture and efficiently colonizing *V. faba* roots, they were applied under greenhouse conditions. At harvest, all plants remained in the vegetative stage. Exposure to 200 µg L^−1^ MCs caused no visible morphological changes in the aerial parts of *V. faba*; however, roots appeared dark brown and were significantly shorter than those of control plants (Fig. 3). Correspondingly, SL, TLN, and RDW were reduced by 18.95%, 16.70%, and 26.40%, respectively, while SDW remained unaffected. Inoculation with rhizobacterial strains alleviated the negative effects of MCs, particularly for TLN and RDW (Fig. 3). For TLN, T4 improved plant performance by 19.51%, followed by C with 9.75%, T10 with 4.87%, and RN7 with no effect. A similar trend was observed for RDW, with improvements of 53.20% for T4, 35.84% for C, 27.53% for T10, and 18.86% for RN7 (Fig. 3).

### Effects of microcystins and rhizobacteria on photosynthetic traits in *Vicia faba*

**Fig. 4:**
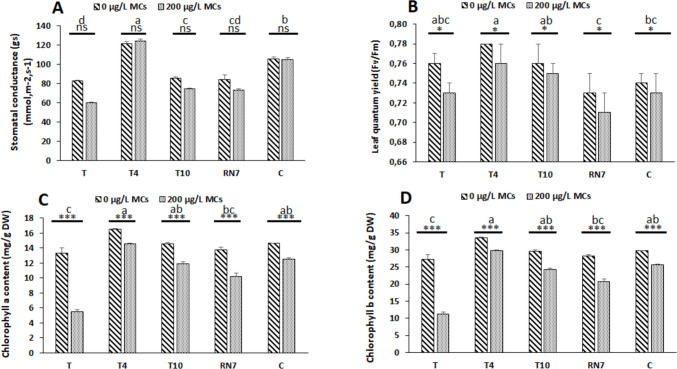
Photosynthetic efficiency evaluated through analyses of **A** stomatal conductance (*g*_*s*_), **B** leaf quantum yield (Fv/Fm), **C** chlorophyll a content in leaves (Chl a), and **D** chlorophyll b content in leaves (Chl b) of *Vicia faba* plants exposed to cyanobacterial crude extract containing 200 µg.L^−1^ of microcystins for 3 weeks under various treatments: T: control (uninoculated plants); T4, T10, and RN7: plants inoculated with rhizobacterial strains; and C: plants inoculated with a consortium of rhizobacterial strains (T4 × T10 × RN7). Mean values (± standard deviations) are based on three replicates. The stars correspond to statistical significance at different levels as follows: ns, not significant; * = *p* < 0.05; ** = *p* < 0.01; *** = *p* < 0.001. Means not sharing the same letter are considered significantly different

Photosynthetic efficiency was evaluated by assessing gs, Fv/Fm, Chl a, and Chl b in *V. faba* plants subjected to MC-contaminated water and inoculated with toxin-tolerant strains (Fig. 4). At a concentration of 200 µg L^−1^ MCs, reductions of 2.59% in Fv/Fm, 24.85% in Chl a, and 24.81% in Chl b were observed, while gs was not affected. Inoculation with rhizobacterial strains improved photosynthetic performance across all parameters (Fig. 4). For gs, T4 displayed the highest increase (71.47%), followed by C (47.16%), T10 (11.72%), and RN7 (10.04%). For Fv/Fm, improvements reached 3.01% with T4 and 1.47% with T10. Chlorophyll contents showed notable enhancements: T4 increased Chl a and Chl b by 64.59% and 63.89%, C by 43.98% and 43.54%, T10 by 40.21% and 39.61%, and RN7 by 26.96% and 26.39%, respectively.

### Influence of microcystin and bacterial inoculation on total nitrogen content and glutamine synthetase activity in *Vicia faba*

**Fig. 5:**
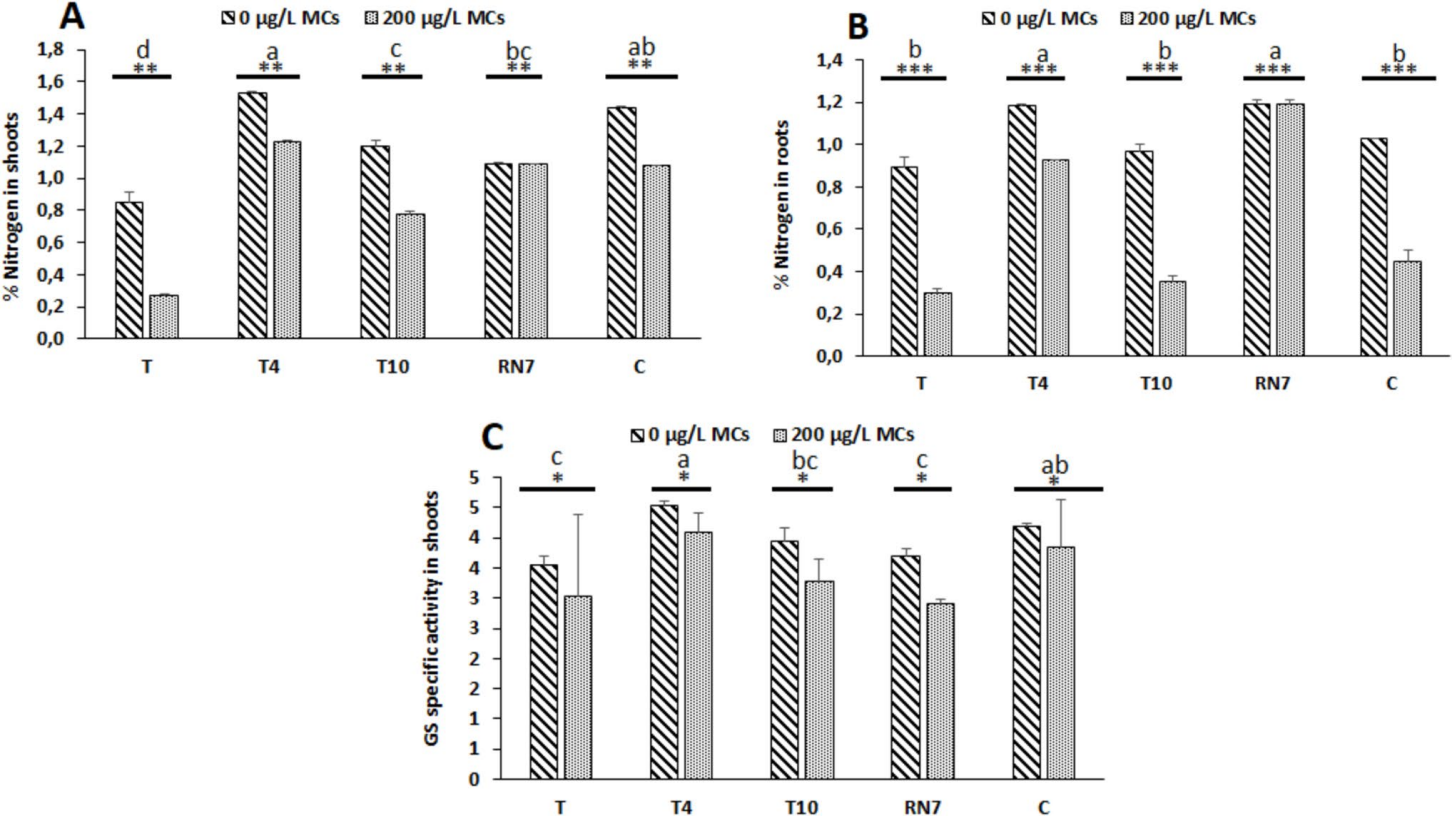
Nitrogen content in **A** shoots and **B** roots and **C** glutamine synthetase activity (GS) in *Vicia faba* plants inoculated with various strains: T (control, uninoculated plants); T4, T10, and RN7 (plants inoculated with rhizobacterial strains); C (plants inoculated with a consortium of rhizobacteria strains T4 × T10 × RN7), and exposed for 3 weeks to cyanobacterial extract containing 200 µg.L^−1^ of microcystins. Mean values (± standard deviations) are based on three replicates. The stars correspond to statistical significance at different levels as follows: ns, not significant; * = *p* < 0.05; ** = *p* < 0.01; *** = *p* < 0.001. Means not sharing the same letter are considered significantly different

Exposure to MCs reduced nitrogen assimilation in *V. faba*, as indicated by lower nitrogen content in shoots and roots and modest decrease in GS activity (Fig. 5A–C). Inoculation with rhizobacteria partly alleviated these negative effects. Strains T4 and T10, as well as the bacterial consortium, enhanced nitrogen content in both shoots and roots, while RN7 primarily improved root nitrogen (Fig. 5A, B). For GS activity (Fig. 5C), differences among treatments were relatively small, although inoculation with T4 and the consortium tended to maintain slightly higher activity compared with the uninoculated control under MC exposure.

### Microcystin bioaccumulationin *Vicia faba* leaves and estimated daily intake following bacterial inoculation

Chronic exposure to cyanobacterial crude extract containing 200 µg L^−1^ of MCs resulted in the bioaccumulation of these toxins in *V. faba* leaf tissues (Table 2). Treatment T4 showed a markedly lower concentration of 1.83 ± 0.09 µg.kg^−1^ compared to the control group, consisting of uninoculated plants irrigated with 200 μg/L of MCs, which exhibited a concentration of 2.86 ± 0.04 µg.kg^−1^, representing a 1.56-fold reduction (Table 2). The EDI for all treatments was below the recommended TDI threshold of 0.04 µg per kg of body weight per day. Consequently, our results indicate a low risk level associated with the consumption of *V. faba* under these conditions (Table 2).

**Table 2:** The total microcystin content in leaves, estimated daily intake (EDI), and health risk quotient (RQ), indicating the extent to which intake exceeds the established tolerable daily intake (TDI) of 0.04 µg.kg person^−1^ d^−1^ for humans, in *Vicia faba* plants assessed following treatment with 200 µg L^−1^ of microcystins for 3 weeks under various treatments: T: control (uninoculated plants); T4, T10, and RN7: plants inoculated with rhizobacterial strains; and C: plants inoculated with a consortium of rhizobacterial strains (T4 × T10 × RN7). Mean values (± standard deviations) were derived from three replicates

**Treatments**	**MCs in water** (μg L^−1^)	**MCs in shoots** **(**μg kg FW^−1^)	**EDI**	**TDI** **(**μg kg^−1^)	**RQ**	**Risk level**
T	0	ND	-	-	-	-
	200	2.86 ± 0.04a	5.24 × 10^−6^	0.04	1.3 × 10^−4^	Low
T4	0	NT	-	-	-	-
	200	1.83 ± 0.09d	3.36 × 10^−6^	0.04	8.39 × 10^−5^	Low
T10	0	NT	-	-	-	-
	200	2.19 ± 0.08c	4.02 × 10^−6^	0.04	1.004 × 10^−4^	Low
RN7	0	NT	-	-	-	-
	200	2.54 ± 0.04b	4.66 × 10^−6^	0.04	1.16 × 10^−4^	Low
C	0	NT	-	-	-	-
	200	2.18 ± 0.04c	4.0 × 10^−6^	0.04	9.99 × 10^−5^	Low

*ND*, not detected; *NT*, not tested

## Discussion

Exposure of *V. faba* to an environmentally relevant concentration of 200 μg L^−1^ MCs significantly induced marked growth inhibition, manifested as reduced SL, TLN, and RDW (Fig. 3). The magnitude of decline was most pronounced in the roots, where dry weight loss was approximately 1.5-fold greater than aboveground parameters, accompanied by visible browning and reduced elongation. This differential sensitivity underscores the root as the primary phytotoxic target, likely attributable to its direct and continuous contact with dissolved toxins, which facilitates local accumulation and cellular injury (Cao et al. 2017). Root dysfunction translated into systemic limitations on canopy development and overall plant performance, consistent with the observed 25% declines in chlorophyll a and b (~ 25%) and a relatively minor reduction in the maximum quantum efficiency of photosystem II (Fig. 4), indicating that MCs propagated to the photosynthetic machinery by destabilizing pigment-protein complexes and impairing electron transport (Arman & Clarke 2021; Melaram et al. 2022). Nitrogen metabolism in *V. faba* was negatively affected by MC exposure, as reflected by reductions in total nitrogen content in both shoots and roots and a modest decline in GS activity (Fig. 5A–C). The slight decrease in GS activity suggests a limited impact on ammonium incorporation into amino acids, potentially constraining protein synthesis and the catalytic efficiency of photosynthetic enzymes (Welten et al. 2020; Yang & Luo 2021). Collectively, these changes suggest a mechanistic connection between root-targeted MC toxicity, systemic metabolic imbalance, and the observed growth inhibition, with nitrogen metabolism representing a sensitive, albeit moderately affected, component in MC-stressed *V. faba*.

In contrast, inoculation with toxin-tolerant rhizosphere bacterial strains possessing growth promoting traits alleviated the inhibitory effects of MCs on *V. faba*, with distinct strain-specific protective profiles reflecting different modes of action. T10 inoculation enhanced root growth and nitrogen content in shoots and roots, supporting nitrogen assimilation and translocation under MC stress (Figs. 3 and 5). Photosynthetic performance was stabilized, with improved stomatal conductance, photochemical efficiency, and chlorophyll content, preserving light-harvesting and carbon assimilation (Fig. 4). These physiological benefits are likely linked to strain-mediated modulation of the plant’s antioxidant systems, which limit oxidative damage and preserve membrane integrity in the presence of MCs (Khan et al. 2021; El-Saadony et al. 2024). Collectively, the data suggest that T10 confers stress resilience not by directly neutralizing the toxin, but by reinforcing core metabolic pathways, enhancing nutrient acquisition, and sustaining photosynthetic function, thereby enabling *V. faba* to maintain growth under MC-induced stress.

*R. leguminosarum* (RN7) exhibited a mitigation pattern that was both selective and root-centered, reflecting its intrinsic biology as a symbiotic-rhizobia. Under MC exposure, RN7 primarily sustained root biomass and nitrogen content (Fig. 3, Fig. 5), thereby reinforcing the organ most directly targeted by toxin stress. This effect is consistent with the strain’s ability to colonize nodules and preserve local nitrogen assimilation, ensuring the continuity of ammonium incorporation and amino acid synthesis at a site where MCs typically disrupt metabolic integrity (Lindström & Mousavi 2019; Schwember et al. 2019; Żebracki et al. 2024). The benefits observed in root nitrogen metabolism extended modestly to aerial tissues, where modest improvements in chlorophyll a and b and a partial stabilization of stomatal conductance were detected (Fig. 4). These shoot-level adjustments are best interpreted as indirect consequences of improved nitrogen fluxes from the roots, since nitrogen availability is indispensable for chlorophyll biosynthesis and Rubisco turnover (Sakuraba 2022; Chai et al. 2024). Such a root-driven buffering mechanism contrasts with the broader systemic protection observed in some non-symbiotic strains, positioning RN7 as a more specialized but strategically effective inoculant. A similar pattern was described by Lahrouni et al. (2016), who showed that rhizobia safeguarded nodulation and nitrogen assimilation in *V. faba* under 100 µg/L MC-LR.

Among all treatments, *A. marplatensis* (T4) consistently outperformed single-strain inoculants and the microbial consortium. T4 markedly improved growth, photosynthetic traits, and nitrogen content while maintaining slightly higher GS activity relative to MC-stressed controls (Figs. 3, 4, and 5), demonstrating a broader and more integrated protective effect than T10 or RN7. Even the consortium, which alleviated MC-induced stress to an intermediate degree, failed to surpass T4 despite its combined functional diversity. Compatibility assays ruled out antagonism, suggesting that the reduced effect of the consortium reflects non-additive trait expression (Table S2), a phenomenon often observed in microbial mixtures under abiotic stress. T4’s superiority likely arises from its combination of rhizo-competent traits, including high IAA production, phosphate solubilization, nitrogen fixation, and HCN production (Table 1). In this research, the method used to evaluate IAA production may overestimate IAA contents of cells due to the detection of other indole compounds (Guardado-Fierros et al. 2024). Consequently, in our results, IAA values may be overestimated (Table 1). This situation should be of no concern since the comparison was done within our selected strains, also, in our screening process, T4 strain represented at least 4 times IAA production as compared to the other strains (Table 1). The highest IAA production (184.5 ± 0.10 µg mL^−1^), in particular, stimulates extensive root proliferation, enhancing nutrient and water uptake while potentially diluting or sequestering MCs away from meristematic tissues, thereby mitigating toxin impact (Viscardi et al. 2016). Previous studies on legume crops have similarly documented the capacity of beneficial bacteria to enhance growth, biomass accumulation, and yield under both stress and non-stress conditions (Shahzad et al. 2008; Stajković et al. 2011; Iqbal et al. 2012; Abdiev et al. 2019; Laranjeira et al. 2021), providing a conceptual framework that supports the observed strain-specific benefits in our experiments. ELISA analyses revealed that T4-inoculated plants accumulated significantly lower foliar MC residues (1.83 ± 0.09 µg.kg^−1^) relative to uninoculated controls (2.86 ± 0.04 µg.kg^−1^) (Table 2), reflecting effective suppression of systemic toxin translocation. These foliar measurements, while derived from non-edible tissues, serve as sensitive early-warning indicators of systemic exposure and rhizobacterial efficacy, with EDI values calculated from leaf MC concentrations remaining below the recommended TDI (Table 2), thereby supporting the strain’s role in mitigating potential risks under MC-contaminated irrigation (Corbel et al. 2016; Cao et al. 2018; Pindihama et al. 2024). The mechanistic basis of this foliar MC reduction remains unresolved but warrants consideration of microbial detoxification. Certain soil bacteria degrade MCs via the mlrA-D pathway, in which mlrA initiates peptide ring cleavage, followed by mlrB/mlrC hydrolysis and mlrD-mediated transport (Tsao et al. 2017; Qin et al. 2019; Wang et al. 2024). Non-mlr pathways, including glutathione conjugation via glutathione-S-transferase, oxidative modification, and other enzymatic or chemical transformations, have been shown to lower MC bioavailability (Haida et al. 2023; Martinez i Quer et al. 2024). Future research should explore the molecular mechanisms for the effects of T4 on *V. faba* under MC stress, to better understand how this strain confers growth promotion and toxin mitigation.

### Originality and significance of the study

These findings advance the field by demonstrating that a single, carefully selected strain (T4) can outperform both single-strain inoculants and microbial consortia under toxin stress, a concept rarely reported in rhizosphere research. This study provides mechanistic insights into how strain-specific traits synergize to enhance plant growth, photosynthesis, nitrogen assimilation, and foliar toxin mitigation, rather than relying on empirical or mixed-inoculum approaches. The originality lies in highlighting strain-specific functional integration and rhizosphere competence as sufficient for broad stress alleviation, challenging the conventional wisdom that consortia inherently provide superior protection. By linking physiological, biochemical, and preliminary food-safety data, this work establishes a publishable framework for both scientific advancement and practical application. Extended trials to pod maturity, field validation, and molecular tracking of rhizosphere toxin degradation pathways are warranted to confirm in situ efficacy and assess potential MC transfer to seeds. Dose–response and repeated-exposure studies will further clarify the boundaries between foliar accumulation and edible tissue contamination, enabling comprehensive risk assessment.

## Conclusion

Our study on *V. faba* plants exposed to MCs revealed significant phytotoxic effects, including growth reduction, chlorophyll loss, and nitrogen assimilation disruption. Although MCs accumulated in plant tissues, the potential human health risks from consuming contaminated fruits remain uncertain, necessitating further investigation. The potential of PGPR, particularly *A. marplatensis* (T4), to mitigate cyanotoxin contamination and reduce toxin accumulation in plants offers promising agricultural management strategies. This strain alleviated MC-induced damage, suggesting sustainable practices for climate change adaptation. Future research should explore the molecular mechanisms for the effects of T4 and evaluate the long-term impact of microbial interventions on crop health and food safety. These findings enhance our understanding of cyanotoxin toxicity and microbial applications for improving agricultural resilience and food security in the face of climate challenges.

## Supplementary Information

Below is the link to the electronic supplementary material.

ESM (DOCX 23.0 KB)

## Data Availability

Not applicable.

## Declarations

### Disclaimer

The funder had no role in study design, data collection and analysis, decision to publish, or preparation of the manuscript.
