# Supplementary material for: Selected rhizobacteria strains improved the tolerance of Vicia faba plants to microcystins contaminated irrigation water and reduced human health risk
Source: Environ Sci Pollut Res Int. 2025 Nov 21;32(47):27101–16. doi: 10.1007/s11356-025-37185-7 (PMC12675700; doi:10.1007/s11356-025-37185-7)
Supplement: Supplementary file 1 — (DOCX 23.0 KB) [file 11356_2025_37185_MOESM1_ESM.docx]

**Supporting Information (SI)**

**Selected rhizobacteria strains improved the tolerance of *Vicia faba* plants to microcystins contaminated irrigation water and reduced human health risk**

**Selected rhizobacteria strains improved the tolerance of *Vicia faba* plants to microcystins contaminated irrigation water and reduced human health risk**

Nadia Elidrissi EL Yallouli^1,2^, El Mahdi Redouane^1,3^, Richard Mugani^1,4^, Lahcen Ouchari^5,6^, Mariana Girão^7^, Maria F. Carvalho^7,8^, Alexandre Campos^7^, Vitor Vasconcelos^7,9,^ Brahim Oudra^1^, Majida Lahrouni^1^, John Poté^2^^[[1]](#footnote-1)^*

### ^1^Biotechnologies and Sustainability of Natural Resources laboratory (Aquabiotech), Cadi Ayyad University of Marrakech, Morocco.

*^2^University of Geneva, Faculty of Sciences, Department F.-A. Forel for Environmental and Aquatic Sciences and Institute for Environmental Sciences, 1211 Geneva 4, Switzerland.

*

*^3^Université de Reims Champagne-Ardenne, Université Le Havre Normandie, INERIS, Normandie Univ, UMR-I 02 SEBIO, Reims, France. Campus du Moulin de la Housse, BP 1039 51687, Reims, CEDEX, France.*

*^4^National Institute of Public Health, Ministry of Health, Avenue Ruvubu, BP 6807, Bujumbura, Burundi^.^*

*^5^Molecular Biology and Functional Genomics Platform, National Center for Scientific and Technical Research (CNRST), 10000 Rabat, Morocco.*

*^6^Microbiology and Molecular Biology Team, Plant and Microbial Biotechnology, Biodiversity and Environment Center, Faculty of Sciences, Mohammed V University in Rabat, 10000 Rabat, Morocco.*

*^7^CIIMAR - Interdisciplinary Centre of Marine and Environmental Research, University of Porto, 4450-208 Porto, Portugal.*

*^8^ICBAS - Institute of Biomedical Sciences Abel Salazar, University of Porto, 4050-313 Porto, Portugal.*

*^9^Department of Biology, Faculty of Sciences, University of Porto, Porto 4169-007, Portugal.*

**S1. Supplementary Tables**

No of Tables = 2 (Table 1S - 2S)

**Table Caption**

**Table S1**. Growth performance of rhizobacterial strains in mineral salt medium supplemented with microcystins as the sole carbon and nitrogen source after 10 days of incubation at 28°C.

**Table S2**. Compatibility test of the three selected rhizobacteria strains.

**Table S1.**

| **Rhizobacterial strains** | **Growth performance** |
| --- | --- |
| T4 | ++ |
| T10 | ++ |
| T2 | ++ |
| T3 | ++ |
| T9 | ++ |
| B9 | ++ |
| F7 | ++ |
| F9 | ++ |
| RN1 | + |
| RN2 | + |
| RN3 | - |
| RN4 | - |
| RN5 | + |
| RN6 | - |
| RN7 | ++ |

The (+/−) indicates positive and negative results respectively. Values are mean of three independent observations. (–) no growth, (+) moderate growth; (++) strong growth.

**Table S2.**

| **Strains** | T4 | T10 | RN7 |
| --- | --- | --- | --- |
| T4 |  | - | - |
| T10 | - |  | - |
| RN7 | - | - |  |

Note: '-' indicates compatibility (no inhibition zone observed).

1. * Corresponding author:

   John Poté, PhD.

   University of Geneva

   Faculty of Sciences

   Earth and Environmental Sciences

   Department F.-A. Forel

   Bd Carl-Vogt 66, CH-1211 Geneva 4

   Switzerland

   Tel: (+41 22) 379 03 21

   E-mail: [john.pote@unige.ch](mailto:john.pote@unige.ch) [↑](#footnote-ref-1)
